# Aplicabilidade de Algoritmos de Aprendizado de Máquina no Diagnóstico de Arritmias – Quanto Tempo Até a Máquina Começar a nos Ensinar?

**DOI:** 10.36660/abc.20250405

**Published:** 2025-08-20

**Authors:** Marcelo Martins Pinto

**Affiliations:** 1 Hospital das Clínicas da Universidade Federal de Minas Gerais Centro de Telessaúde Belo Horizonte MG Brasil Hospital das Clínicas da Universidade Federal de Minas Gerais – Centro de Telessaúde, Belo Horizonte, MG – Brasil

**Keywords:** Arritmia Cardíaca, Inteligência Artificial, Aprendizado de Máquina, Rede Neural, Eletrocardiograma

O eletrocardiograma (ECG), em uso clínico desde o início do século XX, continua sendo uma ferramenta adorada por cardiologistas, clínicos e estudantes de medicina. É indiscutivelmente o instrumento de cabeceira mais importante em cardiologia, essencial para o manejo de arritmias e síndromes isquêmicas agudas, além de orientar o tratamento eletrofisiológico. Ainda assim, os avanços na interpretação de eletrocardiografia foram modestos nos últimos cem anos. Os dispositivos tornaram-se mais portáteis, digitais e capazes de gravações mais longas, mas os princípios interpretativos fundamentais permanecem enraizados nos métodos do início do século XX.^1^ Nos últimos anos, isso foi remodelado pelo surgimento de tecnologias computacionais, especialmente a inteligência artificial (IA). Ferramentas como redes neurais profundas (DNNs) e modelos de aprendizado de máquina (MLMs) estão possibilitando uma nova era na análise de ECG, a eletrocardiografia aprimorada por IA (ECG aprimorado por IA).^2^

Nesta edição do ABC Cardiol, Guimarães do Nascimento et al. relatam uma revisão sistemática de estudos que avaliam a aplicabilidade do ECG com IA no diagnóstico de arritmias cardíacas.^3^ Eles incluíram estudos de diferentes metodologias que abordaram o papel do ECG aprimorado por IA na detecção da síndrome do QT longo (SQTL) (um estudo), intervalo QT corrigido (QTc) (um estudo) e fibrilação atrial (FA) (onze estudos).

Para a detecção de SQTL oculta entre os tipos genéticos 1, 2 e 3, um modelo DNN superou a avaliação tradicional (baseada apenas no QTc) com uma AUC de 0,900 (IC de 95%, 0,876-0,925) versus 0,824 (IC de 95%, 0,79-0,858), respectivamente. O modelo também foi capaz de identificar corretamente o tipo genético com uma AUC de 0,863 (IC de 95%, 0,792-0,934) a 0,944 (IC de 95%, 0,918-0,970), dependendo do tipo genético.^4^

No único estudo que avaliou o QTc *per se* , apenas a precisão da medição foi avaliada, com concordância razoável entre o QTc calculado por IA por um smartwatch e o ECG.^5^ É importante destacar que a aferição automática do ECG é uma meta alcançada desde a década de 1970 com a implantação do software Glasgow e sua consolidação na década de 1980.^6^ A novidade deste estudo reside em mostrar a precisão da medição em diferentes hardwares (o smartwatch) e a possibilidade de auto (ou contínua) medição do QTc.

Entre os artigos que avaliaram modelos de IA na detecção de FA, os métodos foram diversos; no entanto, no geral, os algoritmos de IA e os modelos DNN/ML demonstraram boa sensibilidade e especificidade. É importante destacar que esses estudos relataram a detecção de FA prevalente, com apenas um relatando a análise de ECG em participantes com ritmo sinusal para prever FA, resultando em desempenho modesto (AUM 0,64).^7^ Entre as arritmias cardíacas, a FA tem ganhado particular interesse devido à sua relevância epidemiológica, à diversidade de opções de tratamento e à crescente variedade de estratégias de rastreamento. Embora os benefícios da detecção e do tratamento da FA subclínica ainda sejam discutíveis,^8^ os benefícios do controle precoce do ritmo da FA clínica foram demonstrados.^9^

Este estudo analisa como a IA está sendo aplicada à eletrocardiografia, particularmente na área de arritmias, apresentando desempenho comparável aos métodos tradicionais, e se espera um rápido progresso no curto prazo. No entanto, várias ressalvas devem ser consideradas. A revisão inclui estudos publicados apenas até 2022 e, dado o crescimento exponencial da pesquisa nessa área, estudos importantes podem ter sido perdidos. Por exemplo, em julho de 2023, Yuan et al. relataram o uso de uma rede neural convolucional (CNN) para prever FA a partir de 907.858 ECGs em seis centros de Assuntos de Veteranos, alcançando uma AUC de 0,86 e uma precisão de 0,78.^10^ No mesmo ano, Hygreaal et al. usaram um modelo semelhante em 478.963 ECGs de derivação única de 14.831 adultos mais velhos, alcançando uma AUC de 0,80.^11^ Além disso, Habineza et al. também usaram CNN para prever FA em 1.230.809 ECGs (AUC 0,845).^12^ A validação externa dos resultados dos estudos primários também é um ponto preocupante, visto que a explicabilidade dos modelos de IA, em geral, não é satisfatória. A extração e a avaliação de dados de um único operador também levantam preocupações sobre viés de seleção.

Além da detecção de arritmia, o ECG aprimorado por IA é promissor para outras áreas, como a previsão de insuficiência cardíaca e resultados após eventos cardíacos agudos, o refinamento da estratificação de risco e a possibilidade de permitir relatórios automatizados sem a intervenção do cardiologista.^13 , 14^ No entanto, com essa transformação, surge a necessidade de cautela. A validação científica deve preceder o uso clínico ou comercial generalizado, e questões éticas — como a responsabilização em caso de erro de IA — devem ser abordadas pelas comunidades científica e regulatória.^15^ Ainda assim, a ascensão da IA mantém a eletrocardiografia tão inovadora e intrigante quanto era há mais de um século.
