# Supplementary material for: Aplicabilidade de Algoritmos de Aprendizado de Máquina no Diagnóstico de Arritmias – Quanto Tempo Até a Máquina Começar a nos Ensinar?
Source: Arq Bras Cardiol. 2025 Aug 20;122(8):e20250405. [Article in Portuguese] doi: 10.36660/abc.20250405 (PMC12671721; doi:10.36660/abc.20250405)
Supplement: Translation

# Translation: Applicability of Machine Learning Algorithms in the Diagnosis of Arrhythmias – How Long Until The Machine Starts Teaching Us?

**DOI:** 10.36660/abc.20250405i

**Authors:** Marcelo Martins Pinto

**Affiliations:** 1 Belo Horizonte MG Brazil Hospital das Clínicas da Universidade Federal de Minas Gerais – Centro de Telessaúde, Belo Horizonte, MG – Brazil

**Keywords:** Cardiac Arrhythmia, Artificial Intelligence, Machine Learning, Neural Network, Electrocardiogram

The electrocardiogram (ECG), in clinical use since the early 1900s, remains a beloved tool for cardiologists, clinicians, and medical students. It is arguably the most important bedside instrument in cardiology, essential for managing arrhythmias and acute ischemic syndromes, as well as guiding electrophysiologic care. Still, advances in electrocardiography interpretation have been only modest in the last hundred years. Devices have become more portable, digital, and capable of longer recordings, but core interpretive principles remain rooted in early 20th-century methods.^1^In recent years, this has been reshaped by the rise of computational technologies, especially artificial intelligence (AI). Tools such as deep neural networks (DNNs) and machine learning models (MLMs) are enabling a new era in ECG analysis, the AI-enhanced electrocardiography (AI-enhanced ECG).^2^

In this issue of the *ABC Cardiol* , Guimarães do Nascimento et al. report on a systematic review of studies evaluating the applicability of AI-enhanced ECG in the diagnosis of cardiac arrhythmias.^3^They included studies of different methodologies that addressed the role of AI-enhanced ECG in the detection of long-QT syndrome (LQTS) (one study), corrected QT interval (cQTi) (one study), and atrial fibrillation (AF) (eleven studies).

For the detection of concealed LQTS among genetic types 1, 2, and 3, a DNN model was able to outperform the traditional evaluation (based on QTc alone) with an AUC of 0.900 (95% CI, 0.876-0.925) versus 0.824 (95% CI, 0.79-0.858), respectively. The model was also able to correctly identify the genetic type with an AUC from 0,863 (95% CI, 0.792-0.934) to 0.944 (95% CI, 0.918-0.970), depending on the genetic type.^4^

In the one study evaluating the cQTi *per se* , only measurement accuracy was evaluated, with fair agreement between AI-calculated cQTi by a smartwatch and the ECG.^5^It is important to highlight that automatic measurement of the ECG is a goal achieved since the 1970s with the implementation of Glasgow software and its consolidation in the 1980s.^6^The novelty of this study resides in showing the accuracy of measurement in different hardware (the smartwatch) and the possibility of self (or continuous) measurement of the cQTi.

Among the papers evaluating AI models in the detection of AF, the methods were diverse; however, overall, the AI algorithms and DNN/ML models demonstrated good sensitivity and specificity. It is important to highlight that these studies reported on the detection of prevalent AF, with only one reporting on ECG analysis in sinus rhythm participants to predict AF, resulting in modest performance (AUM 0.64).^7^Among cardiac arrhythmias, AF has gained particular interest due to its epidemiological relevance, diversity in treatment options, and the increasing variety of screening strategies. Although the benefits of detection and treatment of subclinical AF are still debatable,^8^the benefits of early rhythm control of clinical AF have been demonstrated.^9^

This study reviews how AI is being applied to electrocardiography, particularly in the field of arrhythmias, showing comparable performance to traditional methods, and rapid progress is expected in the near term. However, several caveats must be considered. The review includes studies published only up to 2022 and given the exponential growth of research in this field, key studies may have been missed. For example, in July 2023, Yuan et al. reported using a convolutional neural network (CNN) to predict AF from 907,858 ECGs across six Veterans Affairs sites, achieving an AUC of 0.86 and an accuracy of 0.78.^10^That same year, Hygreaal et al. used a similar model on 478,963 single-lead ECGs from 14,831 older adults, achieving an AUC of 0.80.^11^Additionally, Habineza et al. also used CNN to predict AF in 1,230,809 ECGs (AUC 0.845).^12^External validation of the findings of the primary studies is also a point of concern since the explicability of the AI models, in general, is not satisfactory. Data extraction and evaluation from a single operator also raise concerns of selection bias.

Beyond arrhythmia detection, AI-enhanced ECG holds promise for other areas, such as predicting heart failure and outcomes after acute cardiac events, refining risk stratification, and potentially enabling automated reporting without cardiologist input.^13 , 14^However, with this transformation comes a need for caution. Scientific validation must precede widespread clinical or commercial use, and ethical questions — such as accountability in the event of AI error — must be addressed by the scientific and regulatory communities.^15^Still, the rise of AI keeps electrocardiography as innovative and intriguing as it was over a century ago.
